# Effects of β-Lapachone at Non-Toxic and Toxic Concentrations on the mRNA Levels of *XRCC1*, *GADD45A* and *LIG4* Genes

**Published:** 2019-03

**Authors:** Fatemeh SANIE-JAHROMI, Hamideh MAHMOUDINASAB, Mostafa SAADAT

**Affiliations:** Department of Biology, College of Sciences, Shiraz University, Shiraz, Iran

## Dear Editor-in-Chief

Most of anti-cancer drugs have mutagenic, clastogenic, and carcinogenic properties. Studies on oncology nurses and personnel handling cytostatic drugs showed that the frequencies of chromosomal aberrations, sister chromatid exchanges, and micronuclei were significantly increased in personnel handling anti-cancer drugs compared to control group (1). β-Lapachone (β-Lap) is an anti-cancer drug which exerts cytotoxic effect via its induction of ROS generation which ultimately leads to DNA damage (2). Nucleotide excision repair (NER), base excision repair (BER), and non-homologous end joining repair (NHEJ) pathways are very important in genome stability. XRCC1 (OMIM: 194360, an essential scaffolding protein for both long and short patch BER), GADD45A (OMIM: 126335, a growth arrest and DNA-damage-inducible protein) and LIG4 (OMIM: 601837) have important roles in the BER, NER, and NHEJ pathways, respectively. LIG4 efficiently joined single-strand breaks in a double-stranded polydeoxynucleotide in an ATP-dependent reaction (3). LIG4 efficiently joined single-strand breaks in a double-stranded polydeoxynucleotide in an ATP-dependent reaction. LIG4 efficiently joined single-strand breaks in a double-stranded polydeoxynucleotide in an ATP-dependent reaction. Depletion of XRCC1 dramatically sensitized cells to β-Lap (4) and Gadd45a-null mice showed genomic instability (5). β-Lap efficiency can be affected by NHEJ performance (2). Due to anti-cancer property of β-Lap, so it’s occupationally exposure as a public health concern is expected.

To our knowledge there is no study on the effect of β-Lap on the transcript levels of *XRCC1*, *GADD45A* and *LIG4* genes. Therefore the present study was carried out.

SH-SY5Y neuroblastoma cell was cultured in DMEM/F12 enriched with 10% FBS (Gibco), penicillin (100 U/ml, Sigma) and streptomycin (100 μg/ml, Sigma). The cells were seeded at 3 × 10^5^ cells/ml and incubated at 37 °C for 24 h and then cells were treated with β-Lap. Cells were harvested after 24 h and RNA extraction was done. Quantitative real-time PCR and primers specific for the examined genes were described previously (6). 3.2 and 2.0 μm M β-Lap showed about 18% cytotoxicity and no cytotoxicity, respectively. The experiments were done in triplicates. Data were shown as means ± standard error (SE).

**Fig. 1:**
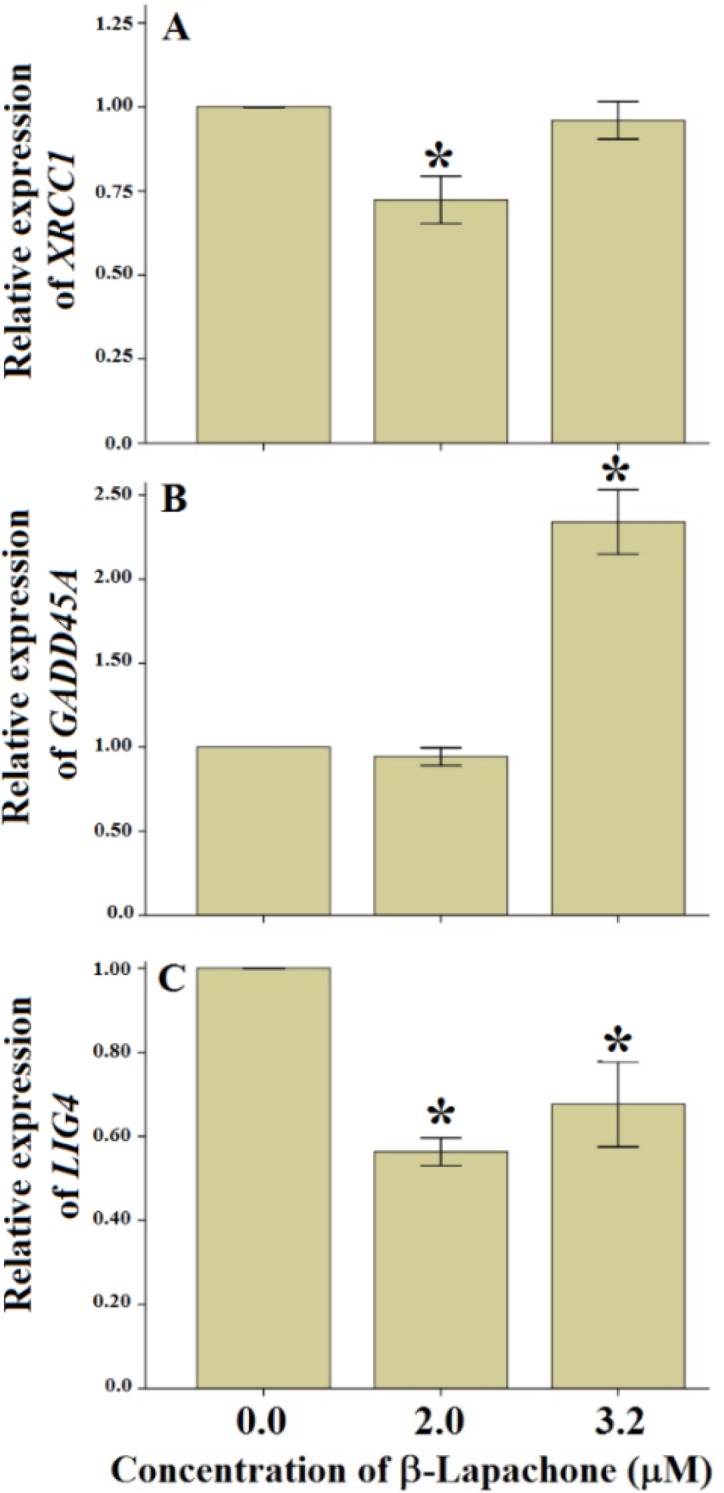
mRNA levels of the *XRCC1* (A), *GADD45A* (B) and *LIG4* (C) genes at two concentrations of β-Lapachone; 2.0 (non-toxic) and 3.2 μM (toxic) concentrations. n = 3, mean ± SE. ^*^P<0.05 all values compared with control cultures using Duncan post hoc test

Figure 1 shows the alteration of mRNA levels of *XRCC1*, *GADD45A* and *LIG4* genes in different treatments. The *XRCC1* mRNA level was significantly decreased at non-toxic concentration. The *GADD45A* mRNA levels did not alter at nontoxic concentration of β-Lap, however, it was significantly increased at toxic concentration of β-Lap, compared with the control culture. The mRNA levels of *LIG4* were significantly decreased at both toxic and non-toxic concentrations of β-Lap. The expression levels of the *XRCC1* and *LIG4* significantly decreased at nontoxic concentrations of β -Lap, cellular DNA repair system cannot repair DNA damages.

We know that nurses of oncology departments and workers handling neoplastic drugs showed higher chromosomal damage compared to control persons (1), which may interpreted by their lower DNA repair capacity due to exposure of non-toxic levels of anti-cancer drugs. Alterations in mRNA levels of DNA repair related genes seem to be a rapid, simple and sensitive method for biomonitoring of effect(s) of occupationally exposure to anti-cancer drugs.

For public health programs, the early detection of alterations may permit the adoption of preventive biological controls such as hygienic improvements in the workplace or the reduction of work hours. Further experiments needs to investigate the effects of other anti-cancer drugs of expression levels of DNA repair genes at non-toxic concentrations.
